# Identification of potential new T cell activation molecules: a Bioinformatic Approach

**DOI:** 10.1038/s41598-024-73003-9

**Published:** 2024-09-27

**Authors:** Mario Morales-Martínez, David Andón-García, Karla Aimee Patiño-Santiago, Jesús Miguel Parga-Ortega, Abrahan Hernández-Hernández, Guillermo Aquino-Jarquin, Genaro Patino-Lopez

**Affiliations:** 1 Immunology and Proteomics Laboratory, Children’s Hospital of Mexico, Mexico City, 06720 Mexico; 2 Department of Cell and Molecular Biology, Karolinska Institute, Stockholm, 17177 Sweden; 3 RNA Biology and Genome Editing Section, Genomics, Genetics, and Bioinformatics Research Laboratory, ’Federico Gómez’ Children’s Hospital of Mexico, Mexico City, 06720 Mexico

**Keywords:** T-cell activation, Signaling Proteins, Immune Response, Lymphocytes, Effector functions, Adaptive immunity, Lymphocytes, T cells, Immunology

## Abstract

T-cell activation is central for the initiation of T cell mediated adaptive immune response and is the result of the close communication between the Antigen Presenting Cell (APC) and the T lymphocyte. Although T-cell activation is currently well understood, and many intracellular pathways are well characterized, nevertheless new players are constantly identified, and this complements the known protein interactome. In this work we aimed to identify new proteins involved in T cell activation. We reviewed and analyzed results of microarray gene expression datasets reported in the public database GEO-NCBI. Using data from GSE136625, GSE50971, GSE13887, GSE11989 and GSE902 we performed different comparisons using R and other bioinformatic tools including GEO2R and we report here upregulated genes that have no previous reports in immune related functions and with potential participation upon T-cell activation. Our results indicate that RND3, SYT10, IgSF6 and PIN1 are potential new T-cell activation molecules.

**Supplementary Information:**

The online version contains supplementary material available at 10.1038/s41598-024-73003-9.

## Introduction

T lymphocytes are at the center of the adaptive immune response, their activation mechanisms are widely studied, many key players and signaling pathways are currently known, for recent comprehensive reviews of the mechanisms and molecules involved in T cell activation see^1–3^. It´s well accepted that productive T cell activation requires three signals^4^ briefly, the first signal is delivered by the interaction of the antigen loaded on the major histocompatibility complex (MHC), on the surface of antigen-presenting cells (APC), and the T cell receptor (TCR), both in CD4 + and CD8 + T lymphocytes. The second signal in helper T cells comes from CD28, which binds to a molecule on the surface of the APC, either CD80 or CD86, this interaction can induce hyper-proliferation of T cells^5^. Cytotoxic T cells require activation signals from co-stimulation molecules such as CD70 and CD137 ^6^. Finally, the third signal requires the action of cytokines, which will determine the fate of T cells, for example, helper T cells can differentiate to Th1 cells by IL-12, Th2 by IL-4 or Th17 by IL-17, IL-6, IL23, each of those will have different effector functions^7^. As a result of those interactions at the interface of the APC and the T cell it will form the immunological synapse where most of the signaling events are initiated, sustained and arguably terminated^8,9^, in this ultra structural arrangement many other molecules participate for example the interaction of LFA-1 with ICAM-I, stabilize T cell/APC interaction^10^, Also as a feedback mechanism TCR signaling induces the activation of Rap1 through ADAP and SKAP55, which promotes the switch to high affinity of LFA-I and ICAM-I^11^. Another molecule with an important regulatory role is CD2, which has both adhesion and co-stimulatory functions reducing T cell activation threshold, this occurs through the phosphorylation of phospholipase C gamma 1 via the Fyn kinase, elevating intracellular calcium levels in response to TCR activation^12^. On the other hand, PD-1, also called CD279, a protein involved in negative regulation of T cell activation has two ligands, PDL-1 and PDL-2, also known as CD274 and CD273, respectively. The interaction of PDL-1 with CD80 generates inhibitory signals^13^. Additionally, PD-1 is a marker of the “exhausted” phenotype, which is characterized by the inability to produce effector cytokines such as IFN-gamma and IL-2, among others. In addition, similarly to CTLA4, PD-1 deficiency in mice produces an autoimmune disease^14^. This pathway has been extensively studied^15^ and reviewed in the context of vaccines^16^, and immunotherapy for cancer^17^.

In addition to the above discussed proteins, there are various elements that play a regulatory role in the context of TCR-MHC interaction, one of them is the actin cytoskeleton, which plays a fundamental role, since the contraction of the cytoskeleton allows T cells to find the APC, as well as generating the transduction force in the TCR^18^. Also, the microtubule network seconds after TCR stimulation promotes the mobilization and polarization of secretory granules for the directed trafficking and secretion of cytokines and cytolytic granules at the IS^19^. Another crucial element in T cell activation is the release of calcium, which is required for full T cell activation and function^20^. Finally, another regulatory mechanism could be the physical forces exerted by actin networks, which are required to maintain stable the TCR-peptide-MHC contact, this is important specially in lymph nodes where the continuous mobility of the T lymphocytes establish initial transient interactions with the APC^21^.

T-cell activation is a highly efficient and specific process that allows to discriminate foreign antigens from self-antigens and is the fundamental mechanism on which the T cell mediated adaptive immune response is based^22^. However, errors in this process can contribute to the development of several pathologies including toxic shock syndrome^23^, autoimmune diseases such as insulin-dependent diabetes^24^, Kawasaki disease^25^ as well as cancer^26^. Therefore, knowing the mechanism in greater detail, as well as investigate the participation of new proteins with functions in the regulation of the activation of T lymphocytes, is of the utmost importance for the identification of new therapeutic targets and the potential development of new immunotherapies, in this work we analyzed new candidate proteins with potential participation in the activation of human CD4 T cells.

## Results

### Identification of potential early T-cell activation candidates

**Fig. 1:**
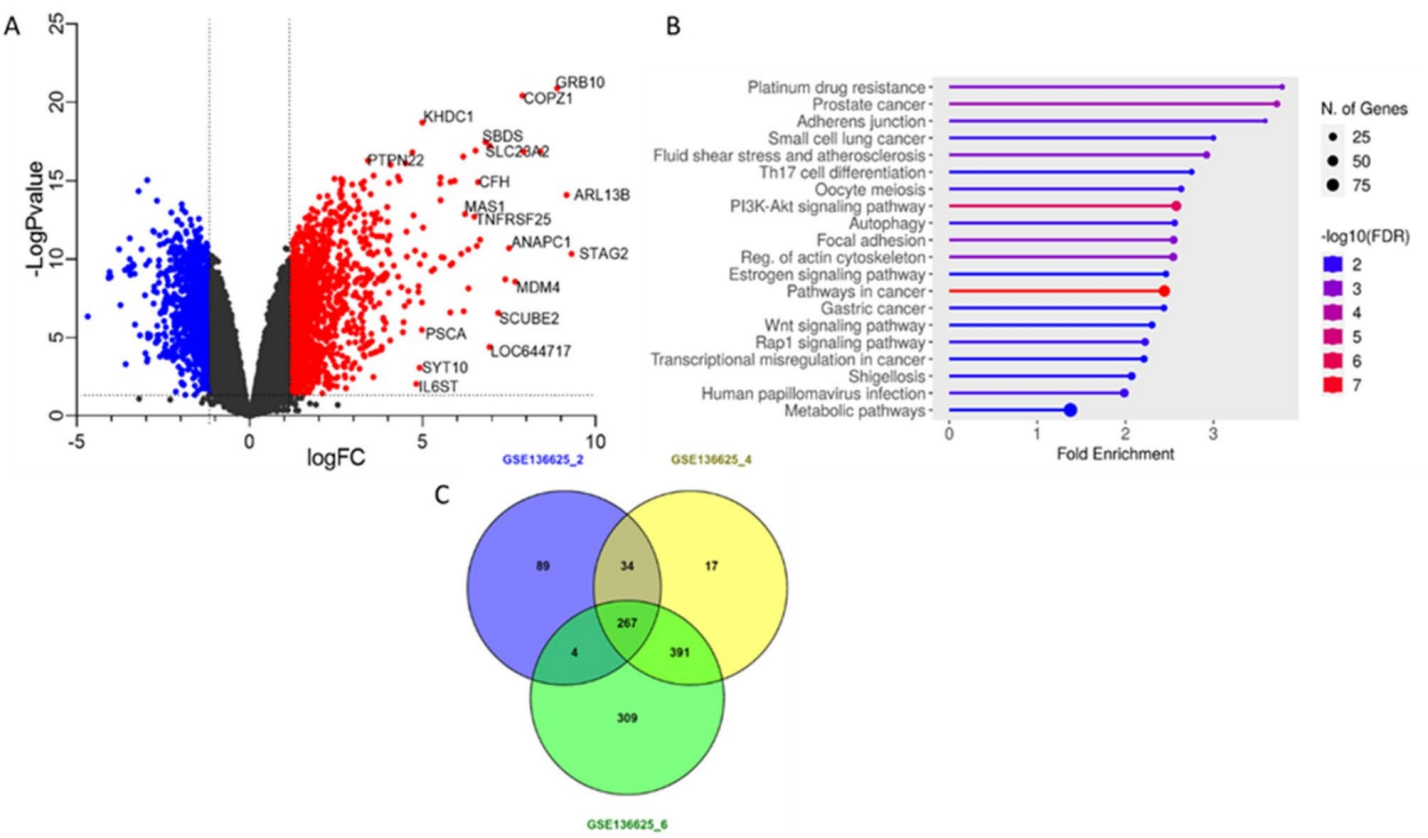
Genes up regulated upon early CD4 T cell activation. **(A)** Volcano plot of total DEG in the GSE136625. **(B)** Graphical pathway enrichment of the genes overexpressed in Fig. 1A. **(C)** Venn diagram of shared up-regulated genes (FC > 1.5 and adjp < 0.05) at 2, 4 and 6 h after activation.

To identify early activation molecules, we focused initially on the expression of different genes upon CD4 + T cell activation at early time points using the GEO data set **GSE136625**, this dataset originally focused in the discovery of miRNAs in T cell activation but also reported data on mRNAs although no formal analysis of these later molecules was done, this dataset comprises the result of gene expression profiles over the initial 24 h of human CD4 + T cell activation^27^ (Available at https://www.ncbi.nlm.nih.gov/geo/ accessed June, 2023). We searched for differentially expressed genes (DEGs), during the initial 6 h post activation, compared to the non-stimulated cells as control. In total we analyzed 2111 genes. The significance versus fold change were analyzed and graphed in the volcano plot (FC > 1.5, adj *p* < 0.05) The result of this analysis indicates genes that were under expressed and overexpressed, blue, and red respectively **(**Fig. 1A**)**. The genes identified as overexpressed were analyzed using the bioinformatic tool ShinyGO 0.77, to identify the principal pathways related to these genes **(**Fig. 1B**)**. We evaluated gene expression at 2, 4 and 6 h using GEO2R (Available on: https://www.ncbi.nlm.nih.gov/geo/geo2r/ Last accession June 2023). The genes with a FC > 1.5 and adj *p* < 0.05 were selected (**Supplementary Table 1**). The results showed 267 genes shared at 2, 4 and 6 h after activation. **(**Fig. 1C**)**. Additionally, we set a more stringent threshold for the adjusted p value to 0.01 and maintained the Fold change value at FC > 1.5. The results showed 181 genes shared at the three different hours (**Figure ****S1**,** supplementary Table 2**). These results were consistent with our initial analysis (Fig. 1). For visualization of the over-expression of those 181 genes **(Suppl Table 2)**, we projected their normalized expression throughout the three different selected time points in a heatmap **(Figure ****S1****)**.

**Fig. 2:**
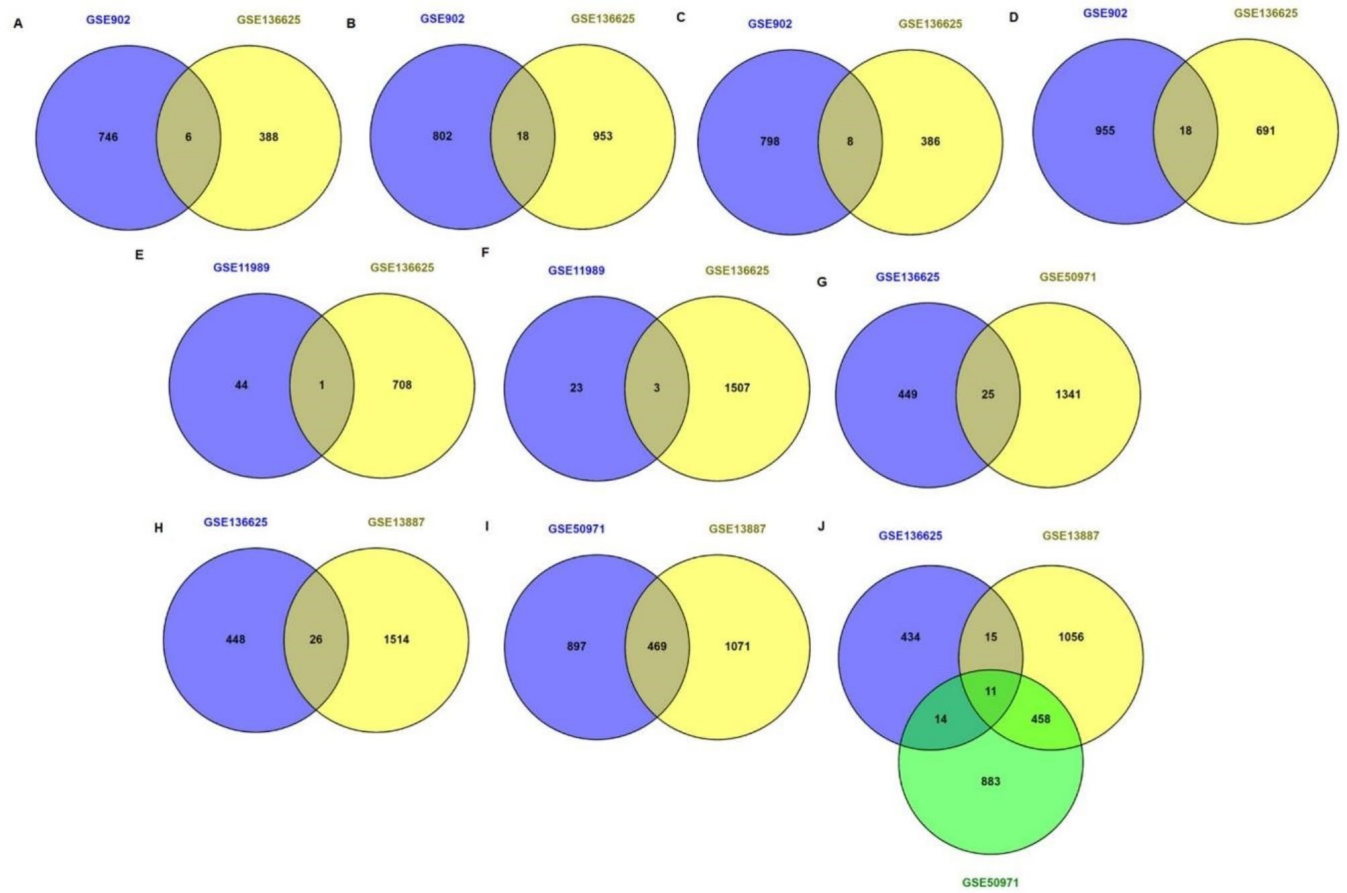
Upregulated genes in GSE136625, GSE902, GSE11989, GSE50971 and GSE13887. Comparison of top gene expression (Log FC > 1.5 and adj *p* < 0.05) shared between GSE902 and GSE136625 at **(A)** 2 and **(B)** 6 h of T-CD4 + PHA blast cells and Jurkat at **(C)** 2 h and **(D)** 4 h. GSE136625 vs. GSE11989 at **(E)** 4 h **(F)** 12 h. At 24 h we compared **(G)** GSE136625 vs. GSE50971. **(H)** GSE136625 vs. GSE13887. **(I)** GSE50971 vs. GSE13887. **(J)** Triple comparison of GSE136625 vs. GSE50971 vs. GSE13887. In all cases the upregulated genes are compared vs. non stimulated or rested cells. The Venn diagrams were prepared with Venny 2.0.2, (Available online at: https://bioinfogp.cnb.csic.es/tools/venny/index.html).

The Jurkat cell line has been widely used to study different aspects of T-cell biology, many signaling pathways have been described in this model^28^, we reasoned that shared molecules between different studies comparing different cells and stimulation conditions might be more likely to be informative, therefore, we compared our previous results of GSE136625 analysis vs. GSE902, which reports T-cell activation from PHA induced blasts and Jurkat T cells stimulated with anti CD3 + anti CD28, we selected time points that coincided with those reported in GSE136625, 2h **(**Fig. 2A**)** and 6 h **(**Fig. 2B**)**, for PHA Blasts and 2h **(**Fig. 2C**)** and 4 h **(**Fig. 2D**)** for Jurkat, we found 6, 18, 8 and 18 genes shared respectively. All these shared genes were reported in the **Suppl Table 3.** Additionally, comparing the datasets **GSE136625** vs. **GSE11989** (Available at https://www.ncbi.nlm.nih.gov/geo/ accessed Jun 2023), which contain data from Jurkat cells activated by PMA/PHA for 4–12 h, we found that at 4 h only one gene shared, RUNX3 (Fig. 2E**)**, while at 12 h we found three shared genes: IER3, BCL2A1 and PRDX1 (Fig. 2F). All the comparisons and shared genes were listed in **Suppl Table 3.** Finally, to identify late activation molecules (24 h) using the same conditions mentioned above (Log FC > 1.5 and adj *p* < 0.05); we compared **GSE136625** versus 3 other datasets which report gene expression from activated T cells. First, we compared **GSE136625** versus **GSE50971** (Available at https://www.ncbi.nlm.nih.gov/geo/ accessed Jun 2023), this dataset report expression data 24 h post activation with PMA/Ionomycin of human lymphoblasts, here we found 25 shared genes **(**Fig. 2G**).** Second comparing with the dataset **GSE13887** (Available at https://www.ncbi.nlm.nih.gov/geo/ accessed Jun 2023), which analyzed expression of genes in total CD3 T cells stimulated 24 h with anti-CD3/CD28 antibodies, we identified 26 shared genes with GSE136625, **(**Fig. 2H**)**. Finally, comparing **GSE13887** and **GSE50971** we identified 469 genes shared **(**Fig. 2I**).** The result of comparing the three datasets showed 11 shared genes. (Fig. 2J, **Suppl Table 3)**. Interestingly when we compared these 11 genes **(**Fig. 2J) versus our early T cell activation molecules from **GSE136625** analysis in Fig. 1, we found 5 shared genes (HSP90AA1, POLR3G, GPR87, SRM, and PTPN11). These proteins, except for PTPN11, which have well known roles in T cell activation^29,30^ represent strong candidates for late activation molecules as they are overexpressed at 24 h regardless of the different stimulus and the different populations studied **(Table ****S4****).** Here we want to highlight that the low number of coincidences could be explained by the different activation protocols, the different microarray platforms (**Table ****S4****)** and by the fact that **GSE136625** would represent de novo T cell activation (primary response) while the other datasets represent effector/memory T cell activation (secondary response).

### Expression pattern of potential candidates for T-cell activation

**Fig. 3:**
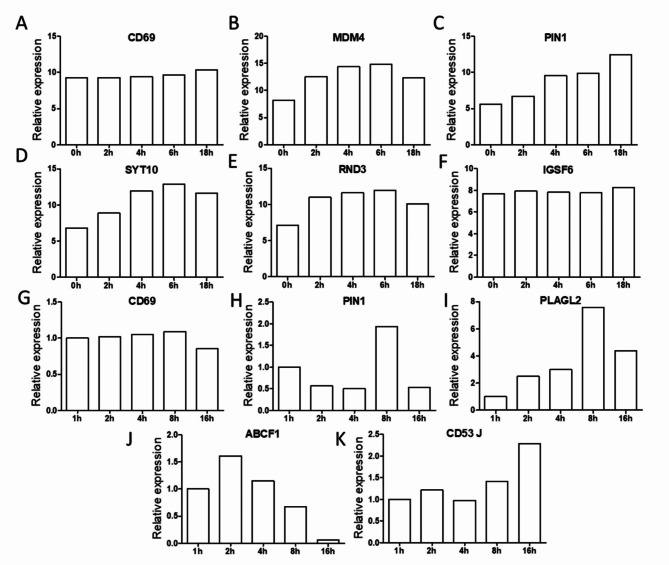
Relative expression over time of genes with potential participation in early T-cell activation from 0 to 18 h post activation from data set GSE136625 (A-F). We present the expression of CD69, MDM4, PIN1, SYT10, RND3, IGSF6 at 0, 2, 4, 6 and 18 h showing the induction and eventual decrease near 18 h for most of them, except for Pin1 that seem to continue increasing. And from GSE902 (G-K). We present the expression of CD69, PIN1, PLAGL2, ABCF1 and CD53 at 1, 2, 4, 8 and 18 h in Jurkat cells showing similar patterns.

To identify the expression pattern of the different genes over time we selected the top 250 over expressed genes in GEO2R from **GSE136625** and then manually inspect the GEO profile for those genes, with special focus of those genes with no known immune related functions (Available on: https://www.ncbi.nlm.nih.gov/geo/geo2r/ Last accession June 2023), briefly, we compared the expression of different genes at resting conditions and at 2, 4, 6, 8, 10, 12, 14, 16 18, 20, 22 and 24 h post activation, we identified genes with an increased expression in the first 2–10 h of activation, in some cases remained increasing, while in others a decrease was detected after 12 h, however, in most cases the expression remained elevated compared to the initial expression at 0 h **(**Fig. 3A-F **)**. This behavior is consistent with our results Figs. 1 and 2 and with the report of Von Andrian et al., which described that in lymph nodes the activation of T lymphocytes was divided into three phases, the first occurred in the first 8 h, where transient contacts take place between T cells and APC and an increase in the expression of activation markers was observed. The second phase consisted of the following 12 h where the contacts were more stable and T cells stopped migrating, and finally after 24 h the T lymphocytes resumed to migrate and began to proliferate^31^. Similar analysis on GEO2R (Available on: https://www.ncbi.nlm.nih.gov/geo/geo2r/ Last accession June 2023) of the dataset **GSE902** showed the expression of different proteins in PHA stimulated CD4 + Blasts and Jurkat T cells, below we present the main candidates with similar kinetics of expression to the previously identified proteins. **(**Fig. 3G-K**).**

### Potential New T Cell Activation Molecules

**Fig. 4:**
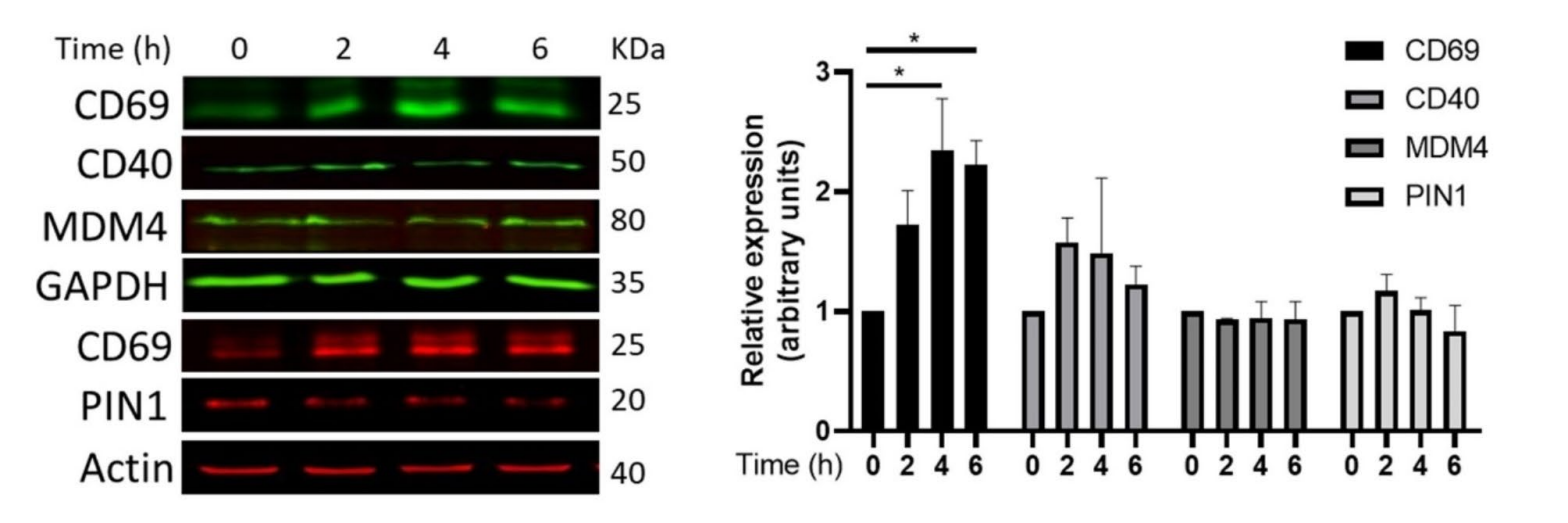
Expression of novel potential early T cell activation molecules. Jurkat cells were stimulated with anti CD3 plus anti CD28 for 0, 2, 4 and 6 h equal amounts of protein (40ug) were loaded and evaluated for protein expression, graphs at the right correspond to the expression at the indicated time (normalized to GAPDH (green) or Actin (red) expression). Representative blots from at least 2 independent experiments are shown, bars correspond to SEM and **p < 0.05.

After a database search comparing the molecules from our different analyses, the expression pattern and what is published for them (**Suppl Table 5**), we selected MDM4, PIN1, SYT10, RND3, RHO, HBZ, IgSF6 and CD53 to determine their expression after in vitro T cell activation, for this initially we evaluated by Western blot using Jurkat cells stimulated with anti CD3 plus anti CD28 antibodies, as control for stimulation we evaluated the expression of CD69 in all cases (Fig. 4). Despite repeated attempts we were not able to get consistent bands for RND3, RHO, HBZ, IgSF6 and CD53. Interestingly we detected CD69 even at resting conditions and was clearly induced after stimulation, also CD40 was readily detected and remained expressed with a tendency to decrease after 18 h, for MDM4, PIN1 and SYT10 we clearly detected their expression although the induction was not obvious.

**Fig. 5:**
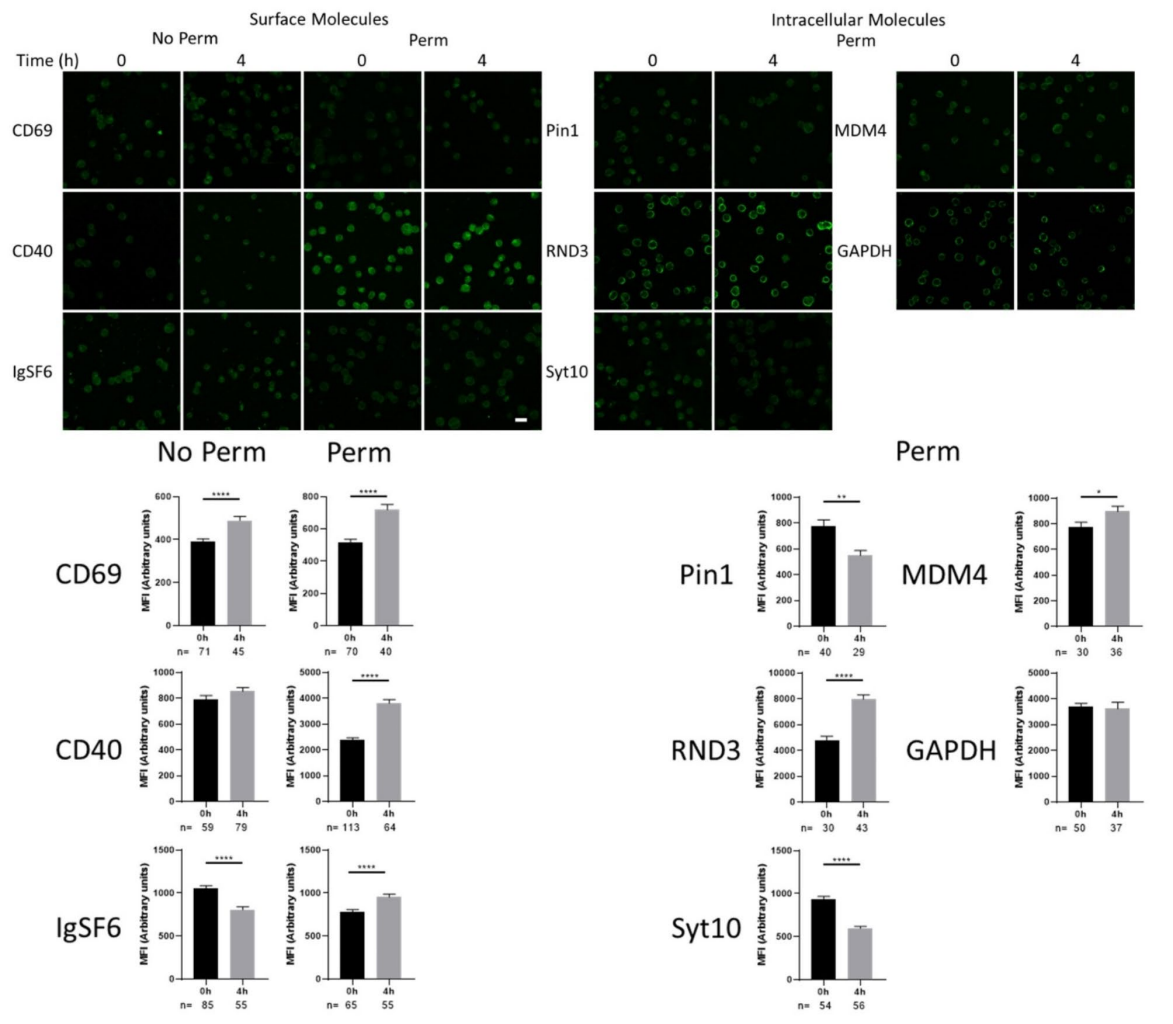
Candidate early activation molecules expression. Representative confocal images for the expression of CD69, CD40, IgSF6, Pin1, RND3, Syt10, MDM4 and GAPDH in resting or 4 h stimulated Jurkat cells with anti CD3 plus anti CD28, surface molecules were evaluated under non permeabilized (to detect surface expression) or permeabilized conditions (to detect potential intracellular pools plus surface expression). Bar represents 10 μm. Below quantification of the fluorescence intensity of different number of cells (indicated below each graph) at resting and 4 h after stimulation, unpaired two tailed t test analysis bars correspond to SEM and *p < 0.05, **p < 0.005, **** *p* < 0.0001.

To determine the expression of the different proteins in whole cells we performed confocal microscopy in anti-CD3/anti-CD28 stimulated Jurkat T cells, we evaluated the expression of surface molecules and intracellular proteins, for the surface molecules we evaluated under non permeabilized and permeabilized conditions to detect possible intracellular pool of the membrane proteins (Fig. 5), here is interesting that the three surface molecules tested (CD69, CD40 and IgSF6 ) gave clear signal at resting conditions and was induced upon stimulation, except for IgSF6 that seemed to decrease in the surface, although the internal pool tended to increase. Also, the expression of RND3 and MDM4 tended to increase, Pin1, and Syt10, tended to decrease and GAPDH didn´t changed after 4 h of stimulation.

**Fig. 6:**
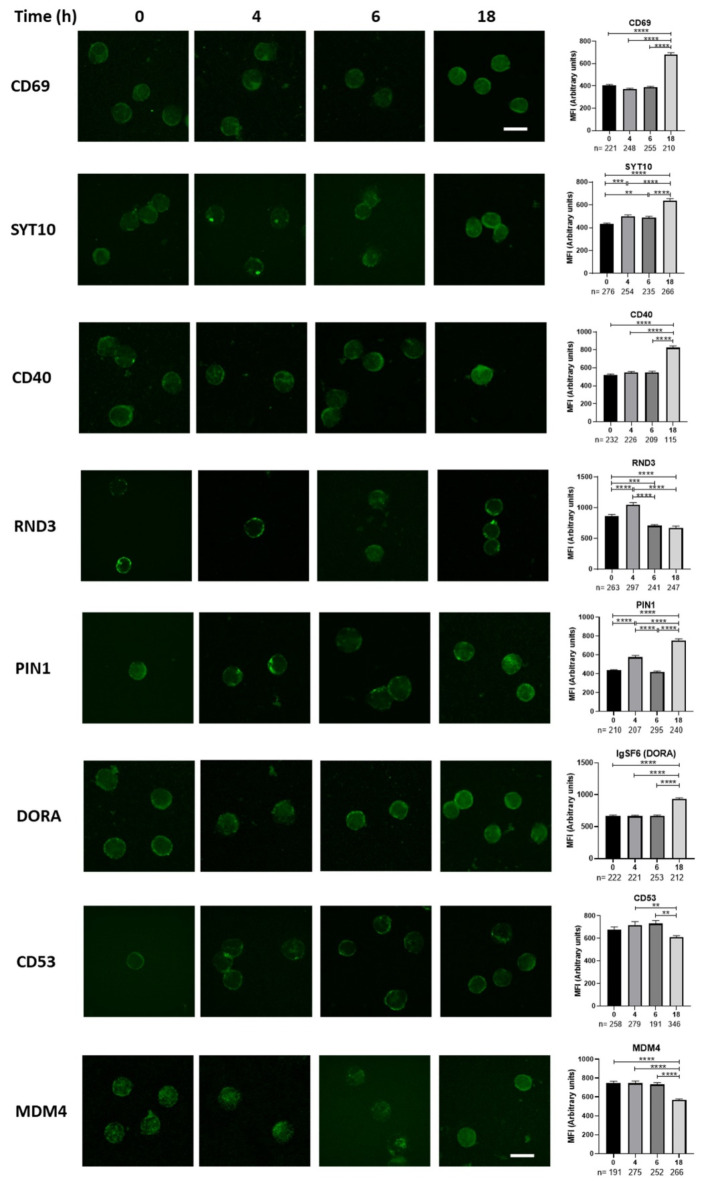
Identification of novel potential activation molecules in primary human T cells. Freshly isolated naïve CD4 T cells were stimulated for 0, 4, 6 and 18 h with anti CD3 plus anti CD28 and processed for immunofluorescence for the expression of CD69, CD40, CD53, IgSF6 (DORA), Syt10, RND3, Pin1 and MDM4, bar correspond to 10 μm. Right panels represent the quantification of 3 independent experiments (1 donor each experiment) with the total number of cells below each graph and time, bars correspond to SEM and **p < 0.01, ***p < 0.001, **** *p* < 0.0001.

Our main goal was to identify novel early activation molecules of primary CD4 T cells; therefore, we evaluated the expression of those candidates in freshly activated naïve CD4 T cells, for this, we left unstimulated or stimulated purified primary naïve CD4 T cells for 4, 6 and 18 h with anti-CD3 plus anti-CD28. Here we found that the expression of Syt10, CD40 and IgSF6, augmented their expression at 18 h post activation, like what we observed for CD69, RND3 increased early at 4 h and decreased after 6 h, maintaining the expression for up to 18 h, Pin1 had a bi modal expression pattern augmented after 4 h, decreased at 6 h and increased again at 18 h. Together these results indicate that Syt10, Pin1, IgSF6 and RND3 might be new early T cell activation molecules (Fig. 6).

## Discussion

Lymphocyte activation has been widely studied, nevertheless its regulation is not fully understood. Hence, identification of new proteins that participate in T Cell activation is essential for a better understanding of this process. Here we analyzed 5 independent datasets from the public database GEO-NCBI reporting T cell activation in CD4 + T-cells and Jurkat using different stimulation methods. Our first approach using the GEO dataset GSE136625 and comparing -Log P value vs. Log FC showed several genes with potential role in T cell activation. To understand the possible functions or pathways involved with those over-expressed genes we selected the most highly significant genes with Log FC > 1.5 and submitted them to the bioinformatic tool ShinyGO 0.77 (available online at: http://bioinformatics.sdstate.edu/go/). Interestingly we identified several pathways related to cancer and in consequence mis-regulation in cell cycle. Nevertheless, only one pathway is related to Th17 differentiation, the relationship between T-cell activation and cancer is a hot topic in the development of immunotherapy against cancer^32,33^ reinforcing the potential of participation of these molecules in the T-cell activation.

A complementary approach to identify potential candidates was the analysis in GEO2R for the genes with the highest expression at early activation times, for this we selected the genes highly up regulated (FC > 1.5) at 2, 4 and 6 h after treatment with anti-CD3/anti-CD28 antibodies. The results showed 400, 720 and 991 genes at 2, 4 and 6 h respectively. Interestingly we identified 267 shared genes at the three times, indicating those genes could be potential regulators of the T-cell activation process. Of note some of the candidates identified in our analyses recently have been demonstrated to participate in T cell activation functions^34–36^.

Those results in primary CD4 T cells were compared with the most upregulated genes in Jurkat, PHA induced Blasts and total CD3 positive cells stimulated for different times with anti CD3 plus anti CD28 and other stimulus like PHA or Ionomycin/PMA, unexpectedly those comparisons showed few shared genes, this could be explained by the difference in the activation method, the nature of the stimulated cells and the analysis platform, however, the low number of coincidences with the different stimuli is consistent with a recent report using single cell transcriptomics where the authors show a clear difference in the transcriptome of primary cells stimulated with anti CD3 plus anti CD28 compared to that of cells stimulated with PMA/Ionomycin^37^. Nevertheless, the comparison at later times (24 h) showed 11 genes shared between three independent datasets GSE136625, GSE50971 and GSE13887, this constitutes genes with potential for late T cell activation molecules. Interestingly when we analyzed the time course for the expression of many of the candidates, we detected a common pattern, that is; an increase from 0 to 8 h, maintained expression from 12 to 18 h and start to decay close to the 24 h time point, this behavior resembles the three phases for T cell activation in lymph nodes described by Mempel. et al. where transient contacts were observed between T cells and APC and an increase in the expression of activation markers was observed in the first 8 h. The second phase occurred the following 12 h where the contacts were more stable and T cells stopped migrating, and finally after 24 h the T lymphocytes resumed to migrate and began to proliferate^31^.

After the bioinformatic approach we evaluated the expression of the selected proteins in vitro, for this we activated primary naïve human CD4 T- lymphocytes and Jurkat. In all cases we compared the expression of the selected protein with that of CD69 as T-cell activation control. Our results indicate that both by Western blot and Immunofluorescence we detected high expression of SYT10, CD40, PIN1, IgSF6(DORA) and RND3 both early (4 h) and late activation (18 h), interestingly the expression was consistent in Jurkat and more importantly in primary recently activated Naïve CD4 lymphocytes indicating these proteins are strong candidates for new activation molecules. One interesting observation we made is the apparent expression of CD69 even in non-stimulated T cells, maybe like a stored pool that is ready to be exported to the surface upon stimulation, also we didn´t detect CD69 as one of the most upregulated mRNAs, this apparent mRNA and protein storage is consistent with reports that indicate that T cells have pools of mRNAs ready to be translated early upon stimulation, but those usually correspond to genes encoding cytokines like IL-2, genes that promote ATP synthesis and metabolic sensing pathways^38,39^.

SYT10 is a calcium sensor for exocytosis of IGF1 in olfactory bulb neurons^40^ and a mediator of neuronal protection against aberrant activity^41^, IgSF6 recently was reported as a critical regulator of ER stress in intestinal Macrophages^42^ and is proposed as a biomarker in lung adenocarcinoma^43^. Pin 1 has been widely studied in Cancer^44,45^ and Alzheimer´s disease^46^. However, knowledge of Pin1 roles in immune cells is limited. One of the most intriguing candidates is RND3 (RhoE) this non classic small GTPase of the Rho family has been widely studied in different processes like promotion of apoptosis in the CNS, negative regulation of ROCK signaling and cytoskeleton remodeling. RND3 knockout mice develop hydrocephalus due to hyperplasia of the ependymal cells, this is due to augmented Notch signaling (NICD), mechanistically RND3 binds and promotes degradation of NICD through the UPS (Ubiquitin Proteasome System)^47^, augmented expression of RND3 in glioblastoma cell line decreases migration and invasion, conversely down regulation promotes increased migration, invasion and proliferation (U251) cells^48^, this is done at least partially by direct interaction of RND3 with Snail, promoting Snail degradation by the ubiquitin-proteasome degradation pathway. Another study demonstrated that lack of RND3 expression causes accumulation of lysosomes, lipid droplets and irregular mitochondria^49^, downregulation of Rnd3 expression allows the induction of mitochondria fission by the RhoA/ROCK I pathway which, in turn, inhibits oxidative phosphorylation and activates glycolysis. Finally, RND3 overexpression inhibited VSMCs (vascular smooth muscle cells) migration and proliferation in SHR (spontaneous hypertensive Rats), RND3 overexpression in VSMCs reduced NAD(P)H oxidase (NOX) activity, NOX1 and NOX2 expression, mitochondria superoxide generation, and H2O2 production in SHR^50^. In Lymphocytes is not known what functions RND3 might control, further studies in our lab will address this issue.

Another protein of interest is CD40, a member of the TNF receptor family who is well known costimulatory protein classically expressed by the antigen presenting cell and whose functions are well described for immunoglobulin switching in T-B cooperation, amplification of inflammatory responses, DC maturation, among others^51–53^, interestingly there are several early reports describing functions of CD40 in T cells^54,55^, also different groups reported expression of CD40 in pathogenic CD4 T cells in type 1 diabetes^56,57^, further studies will define the specific functions and relevance of the expression of CD40 in Jurkat and primary hCD4 T cells.

One limitation of our work is that only upregulated genes were considered, future work should include down regulated genes that might give a more complete picture of the elements that are controlling early activation of T cells, another limitation is that we evaluated the expression of several candidates however we didn´t evaluate the functions that those proteins are playing in the activation mechanisms and eventual effector functions of CD4 T cells.

In summary, despite the vast current knowledge of T-cell activation, several reports have been accumulated through the years adding proteins and molecules with roles as regulators or pathway effectors, revealing that the process is more complex than we think. Certainly, connecting the dots between the known pathways with new players will help to overcome current roadblocks in the efficacy of CAR-T and checkpoint blockade therapies. As described in this work using the datasets GSE136625, GSE50971, GSE13877, GSE11989 and GSE902, deposited in the public database GEO-NCBI, we identified RND3, CD40, SYT10, IgSF6 and PIN1 as potential novel early T-cell activation molecules.

## Methods

### Bioinformatic analysis

The analysis of the datasets was performed initially applying linear models for microarray data in R (limma_3.52.4 in R version 4.2.1) we used Log Fold Change (FC) > 1.5 and adj *P* < 0.05 this allowed us to identify an initial list of gene candidates to T-cell activation molecules. Further analyses were conducted using GEO2R. For this we selected default parameters, except for the use of Limma and systematic assignation of the specific time point of stimulation (query) first and control in second place (subject) and resetting after each analysis. To select the candidates, in another analysis where we compared different cell types and stimuli, we used Log Fold Change > 1.5 and adj *P* < 0.01. These parameters although gave low number of coincidences allowed us to improve our confidence in the results, furthermore CD69 that was used as the bona fide activation marker was detected almost always at the end of the selected values (was never detected in the top 100 candidates). In all our analyses we downloaded the full table, and all the data were pasted in Excel where we custom sort by Log FC and adj P values, for all our analyses we selected only the up-regulated DEGs.

### Cell culture

Jurkat (E6-1) (Kind donation of Dr. Stephen Shaw, NCI, NIH) were cultured in RPMI 1640 (CORNING) medium with L-Glutamine and 25mM HEPES, mented with; 10% Fetal Bovine Serum (FBS) (GIBCO), 1% Non-Essential Amino Acids 100X (CORNING), 1% Sodium Pyruvate 100mM (CORNING) and 1% Antibiotic-Antimicotic (BIOWEST) 1%. At 37 °C and 5% CO2. To ensure homogeneous CD3 expression we sorted positive cells and performed all our experiments with those freshly selected cells, briefly, we stained 1 × 10^6 cells contained in 200µL of culture medium, with 2.5 µL of mouse anti-hCD3 (130-093387, Mitenyi Biotec) incubated for 30 min at 4 °C protected from light, then washed at 1500 rpm/5min and resuspended in 200 µL of culture medium, then we added 2.5 µL of goat anti mouse anti-IgG secondary antibody labeled with Alexa Fluor488 (ab150113, abcam), incubating again under the above conditions. The cells were washed by resuspending them in 1 mL of culture medium for identification, classification and selection using the “BD Influx ™ cell sorter”.

### Purification of Naïve CD4 (+) T lymphocytes

This study was conducted according to the guidelines of the Declaration of Helsinki, all human samples were authorized by written informed consent and approved by the Research, Ethics and Bio safety Committee of the Hospital Infantil de Mexico, Federico Gómez protocol HIM/2022/015. Briefly, 15 mL of peripheral blood from healthy donors (3) was collected by venipuncture in vacuum collection tubes with K2 EDTA anticoagulant “Vacutainer, Becton Dickinson”, the blood was diluted 1:1 with sterile 1X PBS supplemented with 2% FBS and carefully poured in the form of a layer over 15 mL of Lymphoprep “Stemcell technologies”. Subsequently, we centrifuged at 800 g for 25 min collecting the mononuclear cells at the plasma-Lymphoprep interface. The recovered mononuclear cells were resuspended in 2% PBS-FBS up to a volume of 50 mL and washed at 300 g/5min to resuspend in PBS 1x with 2% FBS or culture medium and initiate the naïveNaive CD4(+) T cell isolation protocol. Briefly, 1 × 10^7 mononuclear cells suspended in 40 µL of PBS pH:7.2 with 0.5% BSA and 2mM EDTA. 10 µL of biotin-antibody cocktail from Naive II CD4 + T cells “Miltenyi Biotech” was added to the suspension and mixed and incubated for 15 min at 4 °C. Then 30 uL of buffer and 20 uL of microbead Cocktail II “Miltenyi Biotech” was added, mixed, and incubated for 10 min at 4 °C. The final volume was brought to 500 uL. The column (MACS Columns^®^, Miltenyi Biotec) already placed in the magnet was rinsed with 3 mL of buffer and after that, the cell suspension was deposited in it, recovering the eluate with the enriched Naïve CD4 (+) lymphocytes (CD45 RO-, CD25- cells), the attached cells were washed 2 times with 500 µL of buffer without detaching the column from the magnet. After combining the 3 fractions, the collected lymphocytes were washed at 300 g/5min and resuspended in supplemented RPMI culture medium to initiate activation experiments. 

### Activation of Jurkat cells and primary human naïve CD4(+) T lymphocytes

For T cell activation assays, we selected 0, 4, 6 and 18 h, for this, 2 × 10^6 cells/ml suspended in complete medium were deposited in a 12 well plate. Each well, except the control cells was treated with 1ug/ml of anti-CD3 (130-093-387) and 1ug/ml of anti-CD28 (130-093-375) antibodies (Miltenyi). After the activation time, the reaction was stopped by adding 1 mL of ice-cold PBS, the cells were washed at 300 g for 5 min, then resuspended in PBS and processed for WB or Immunofluorescencen time, the reaction was stopped by adding 1 mL of ice-cold PBS, the cells were washed at 300 g for 5 min, then resuspended in PBS and processed for WB or Immunofluorescence.

### Western blot

Cell lysates were obtained by lysing the cells in RIPA buffer “Abcam” with protease inhibitor cocktail “Pierce, Thermo scientific”, incubated for 30 min on ice, mixed by vortex mixing for 2 min and centrifuged at maximum speed for 15 min at 4 C, then we quantified protein by the “DC Protein Assay kit, BIORAD”. After, we resolved 40 ug of denatured total cell lysates in 12% or 15% acrylamide gels, transferred to 0.22 μm nitrocellulose membranes in a “Trans-Blot Turbo Transfer System, BIORAD )” for 10 min. Membrane blockade was performed with 5% nonfat milk in PBS 1X with 0.1% Tween20 (PBS-T) overnight and the primary antibodies used were: α-CD69 (1:1000, Abcam, Ab181602), α-CD40 (1:1000, Abcam, Ab13545), α-SYT10 (1:500, Novus Biologicals, NBP2-85861), α-RND3 (1:500, Abnova, H00000390-MOI), α-DORA (1:500, Euro Scientific, DDX0220P100), α-GAPDH (1:10000, Abcam, Ab233396), α-CD53 (1:1000, Novus biologicals, NB500393), α-PIN1 (1:1000, RyD Systems, MAB2294), α-HBZ (1:1000, R&D Systems, MAB7708), α-MDM4 (1:1000, Novus Biologicals, NB100556), all the primary antibodies were incubated 1 h at room temperature, washed three times for 5 min in PBS-T, after, we incubated the membranes with the corresponding secondary antibodies for 1 h in PBS-T. Anti mouse 1:10000, Invitrogen, A21058 and anti-rabbit 1:10000, Life technologies, A21109, labeled with Alexa Fluor^®^ 680 were incubated at room temperature protected from the light and after 3 washes with PBS-T and a final wash with PBS the blots were recorded using the “Odyssey CLx Imager, LI-COR”.

### Immunofluorescence

After T Cell activation the cells were adjusted to 1.5*10^6^/ml in 2% FBS in PBS, then 10ul (1.5 × 10^4^) were deposited in a glass slide, let stand for 10 min and then added 20ul of 4% formalin to fix the cells for 10 min, after 3 washes of 5 min with PBS 1X the cells were permeabilized with 0.5% triton X-100 in PBS for 25 min, after permeabilization the cells were washed and incubated for 1 h in a humid chamber with blocking buffer (2% FBS in PBS). Then the cells were washed 3 times with PBS and incubated for 1 h with primary antibody: α-CD69 (1:20, Abcam, Ab181602), α-CD40 (1:20, Abcam, Ab13545), α-SYT10 (1:20, Novus Biologicals, NBP2-85861), α-RND3 (1:50, Abnova, H00000390-MOI), α-DORA (1:20, EuroScientific, DDX0220P-100), α-GAPDH (1:50, Abcam, Ab233396), αCD53 (1:20, Novus biologicals, NB500393), α-PIN1 (1:20, R&D Systems, MAB2294), α-MDM4 (1:20, Novus Biologicals, NB100556) dissolved in 1X PBS with 2% FBS. After that, 3 washes were performed and then incubated for 1 h with the secondary antibodies α-mouse (1:300, Jackson Immuno Research, 115-454062) and α-rabbit (1:300, Jackson Immuno Research, 111-545-045) labeled with Alexa Fluor^®^ 488, at the end 3 washes with PBS and one more with distilled water, then 15 uL mounting medium “VECTASHIELD^®^ Antifade with DAPI (H-1200) was added, Vector Laboratories” to cover with a slide and seal with commercial nail polish, the images were acquired on a Leica TCS-SP8x confocal microscope, equipped with white laser and high sensitivity detectors (HyD) using a 63X oil immersion objective. For all our experiments we adjusted the gain to the brighter signal making sure no saturation was detected, after that, all images were acquired with identical settings, selecting the field of observation in bright field (blind for the fluorescent channel) and acquiring 3–6 fields per experiment to make sure at least 50 cells per condition/molecule were recorded.

### Immunofluorescence analysis

The “ImageJ” program (Fiji version) was used as digital processing and analysis software. For the analysis the files were open with bioformats; selecting to view each in “Hyper stack” mode, using ROI Manager we activated the “Display ROIs” option, the files were opened individually and selected the colored option. Then we selected the corresponding image in the Alexa 488 channel, we visualized all the stack and manually selected the image around the center where most of the signal was detected, we copied that image and applied a “Gaussian blur” with a value of 1. After this, we inverted the color of the image and activated the “Threshold” command to select all the individual cells, generating a “Binary Mask” were the cells well-defined are circled and now called “particles”, those particles were uniformly labelled by the “Fill Holes” command and to separate possibly concatenated cells we used the “watershed” command. Then, the generated “particles” were analyzed showing the results in the ROI manager, we used for all our analyses integrated density “IntDen” to generate the graphs using GraphPad Prism 8.0.1.

#### Statistical analysis

All the results are from 3 independent experiments unless otherwise specified, we used one way analysis of variance (ANOVA) with Tukey for multiple comparisons and two tailed unpaired t test for comparison of two groups using GraphPad Prism 8.0.1 and *p* < 0.05 was considered as significant.

## Electronic supplementary material

Below is the link to the electronic supplementary material.

Supplementary Material 1

Supplementary Material 2

Supplementary Material 3

Supplementary Material 4

Supplementary Material 5

Supplementary Material 6

Supplementary Material 7

## Data Availability

All data generated or analyzed during this study are included in this published article (and its Supplementary Information files).
