# Supplementary material for: Identification of potential new T cell activation molecules: a Bioinformatic Approach
Source: Sci Rep. 2024 Sep 27;14:22219. doi: 10.1038/s41598-024-73003-9 (PMC11436975; doi:10.1038/s41598-024-73003-9)
Supplement: Supplementary file 1 — Supplementary Material 1 [file 41598_2024_73003_MOESM1_ESM.pdf]

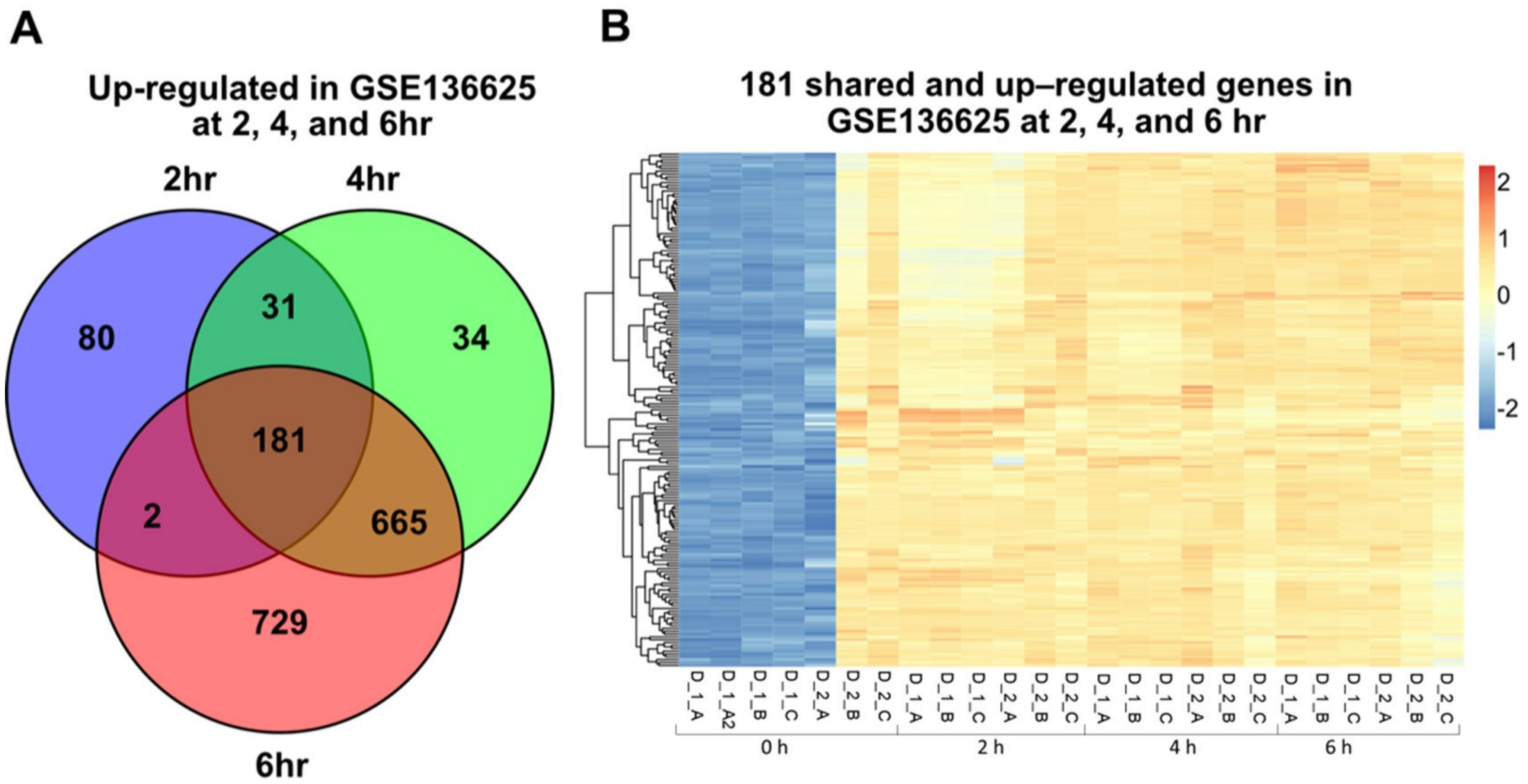

Suppl Figure 1. Up-regulated genes in GSE136625 dataset. A) Venn diagram of shared up-regulated genes (FC>1.1 and p<0.01 at 2, 4 and 6 hours after activation. B) Heatmap displaying the relative expression of the 181 shared and up regulated (from panel A) across the different individuals at the three evaluated data points from the GSE136625 dataset.
