# Supplementary material for: Identification of potential new T cell activation molecules: a Bioinformatic Approach
Source: Sci Rep. 2024 Sep 27;14:22219. doi: 10.1038/s41598-024-73003-9 (PMC11436975; doi:10.1038/s41598-024-73003-9)
Supplement: Supplementary file 7 — Supplementary Material 7 [file 41598_2024_73003_MOESM7_ESM.pdf]

Full Blots related to figure 4 (Up)

In the left is the specific molecule, in the right the corresponding GAPDH loading control (The membranas were stripped and re-probed for GAPDH)

The orange square highlights the region shown in the figure

CD69

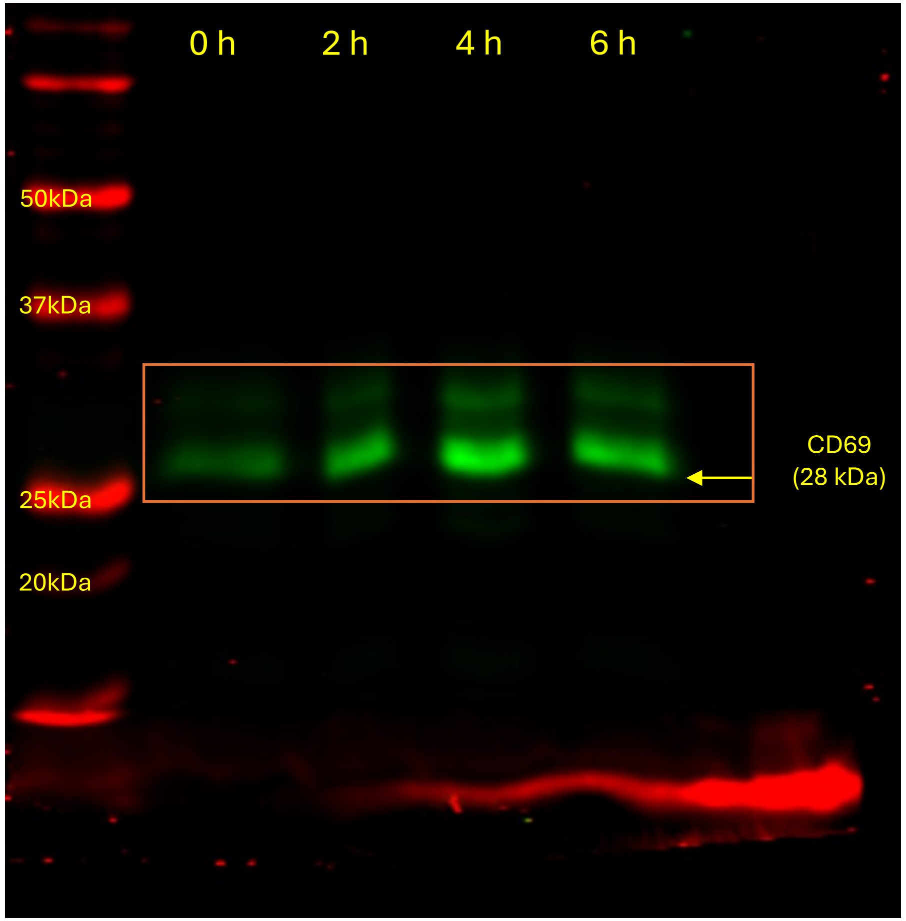
0 h
2 h
4 h
6 h
50kDa
37kDa
25kDa
20kDa
CD69
(28 kDa)

GAPDH

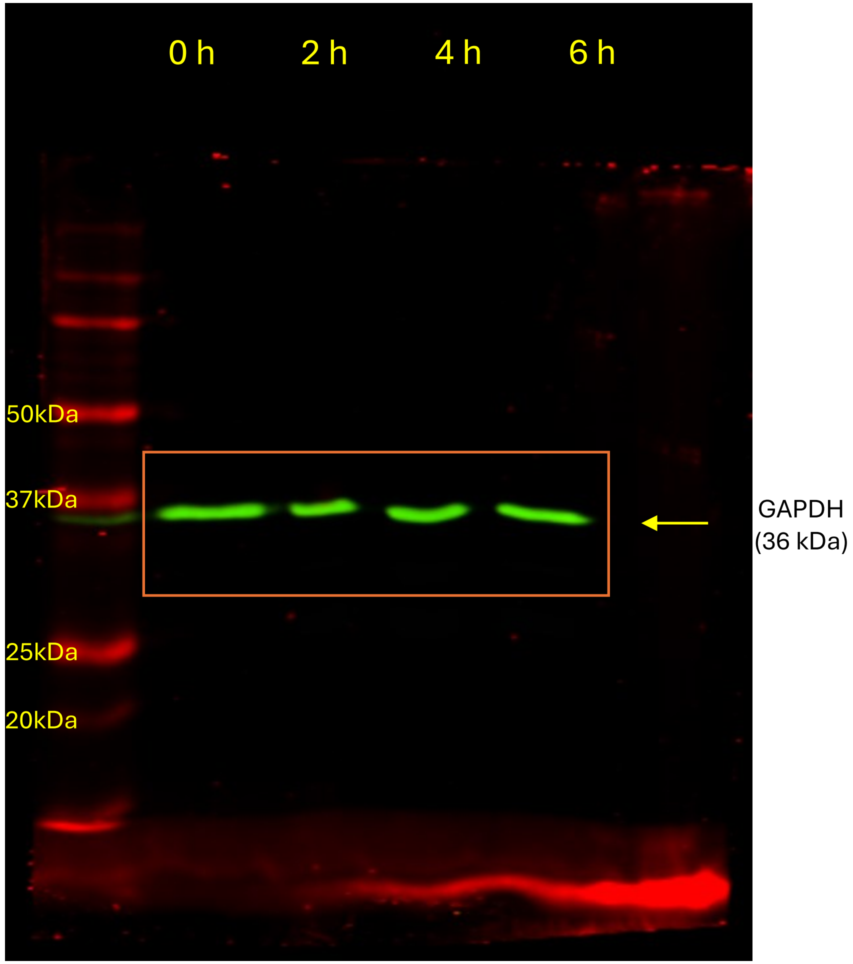
0 h
2 h
4 h
6 h
50kDa
37kDa
25kDa
20kDa
GAPDH
(36 kDa)

CD40

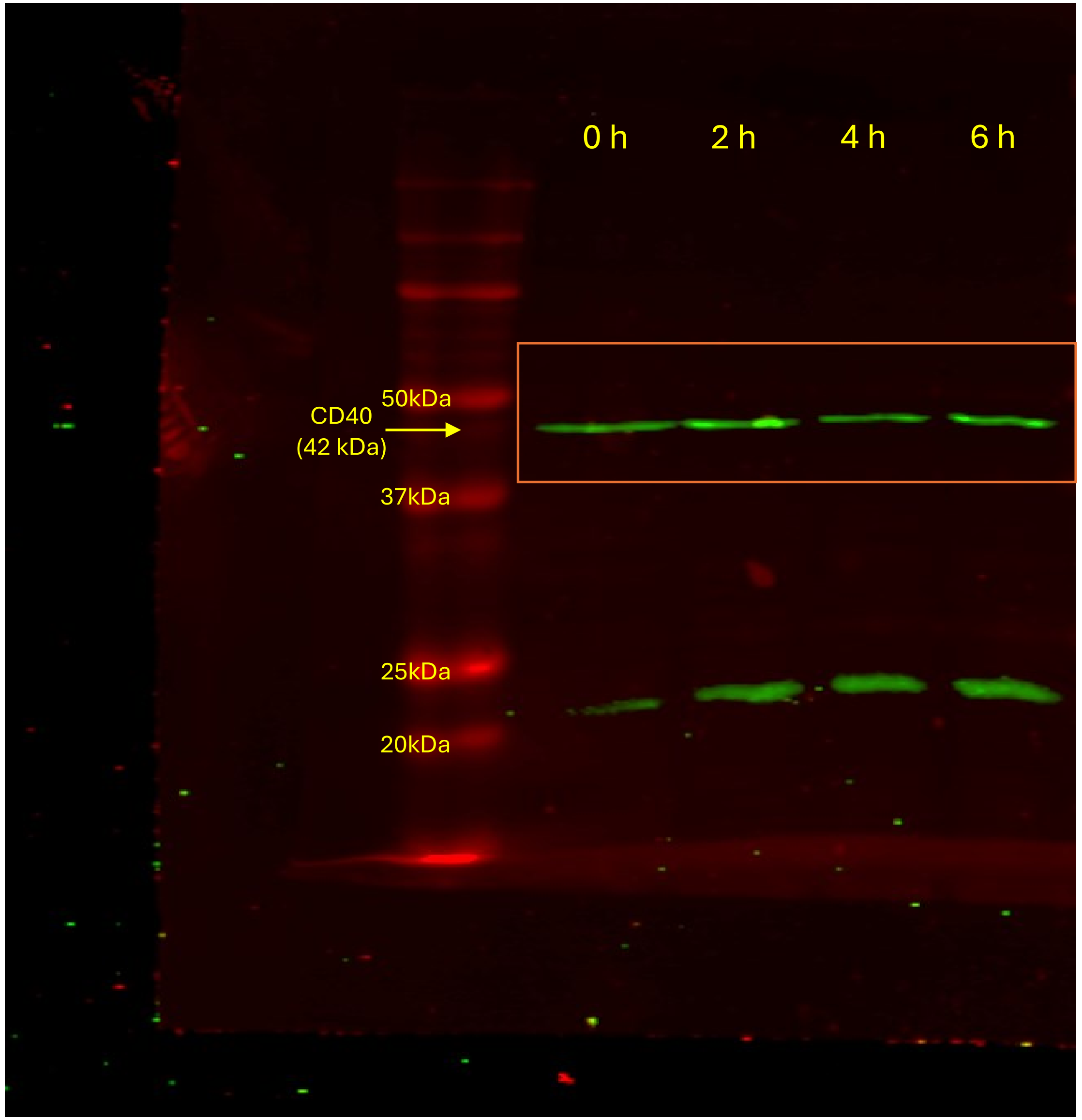
0 h
2 h
4 h
6 h
50kDa
CD40
(42 kDa)
37kDa
25kDa
20kDa

GAPDH

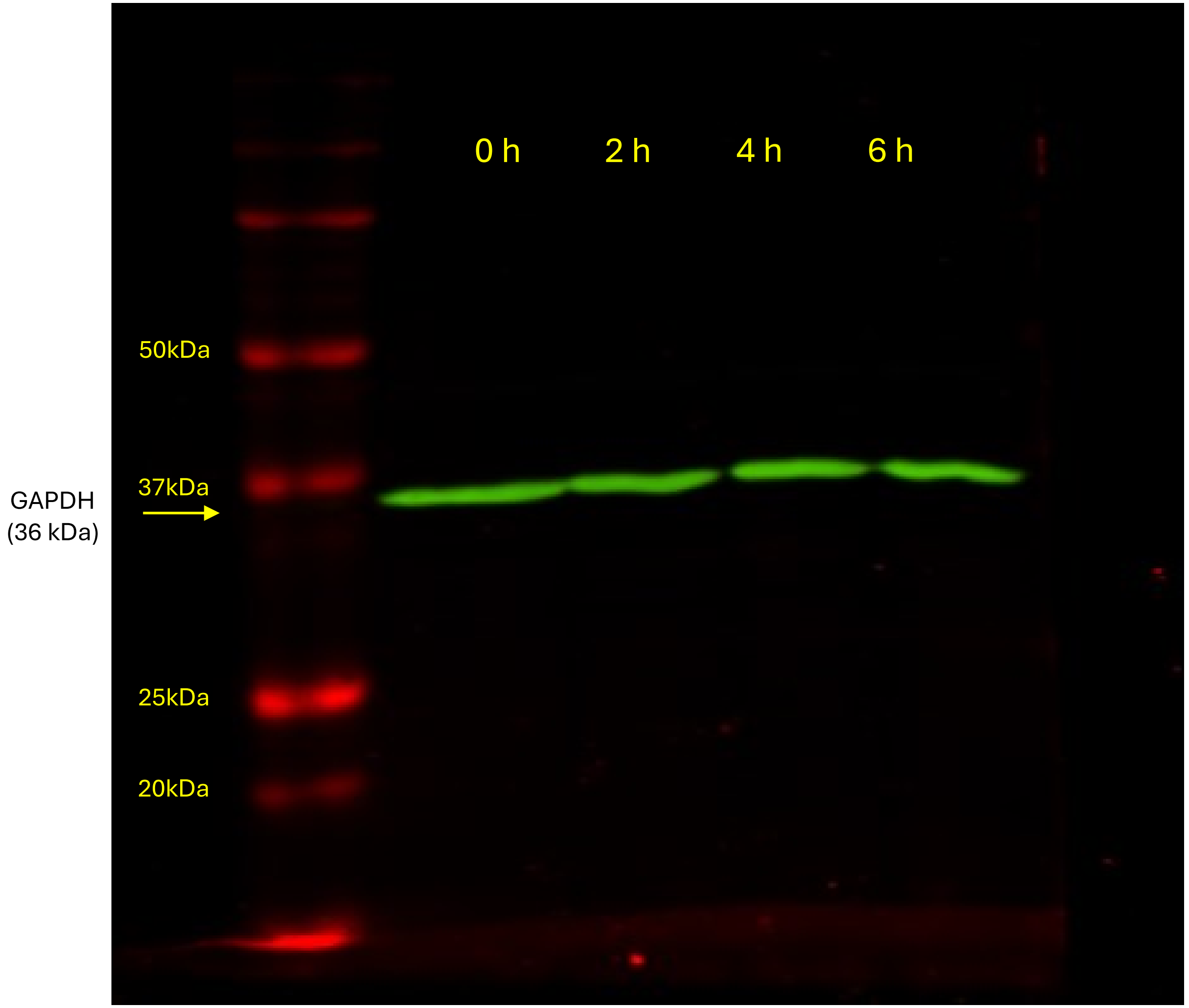
0 h
2 h
4 h
6 h
50kDa
37kDa
GAPDH
(36 kDa)
25kDa
20kDa

MDM4

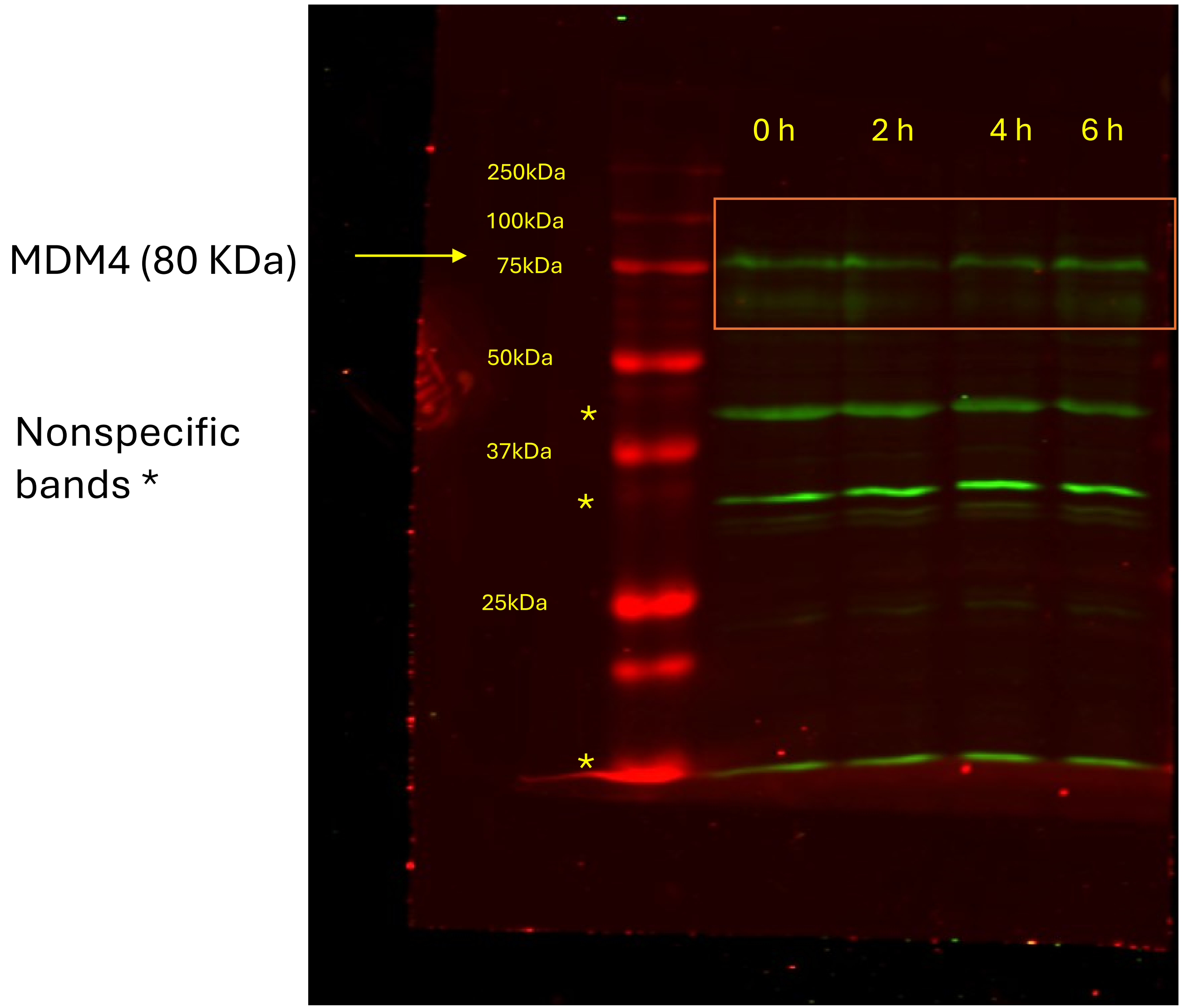
MDM4 (80 KDa)
0 h
2 h
4 h
6 h
250kDa
100kDa
75kDa
50kDa
37kDa
25kDa
Nonspecific
bands *
*
*
*

GAPDH

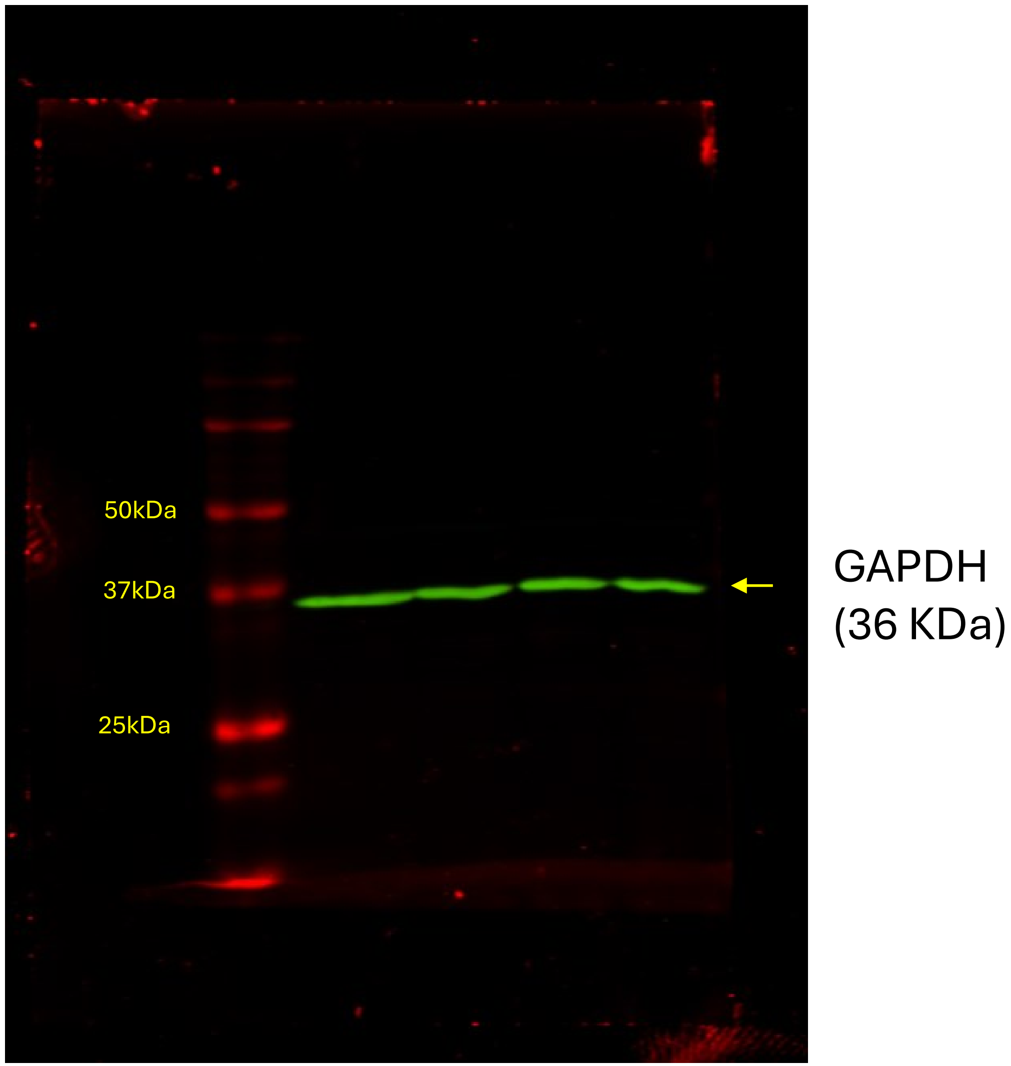
50kDa
37kDa
25kDa
GAPDH
(36 KDa)

Full Blots related to figure 4 (bottom)

The full blot for the specific molecule is shown, in this experiment we show the blot for actin from a stripped membrane

The orange square highlights the region shown in the figure

CD69

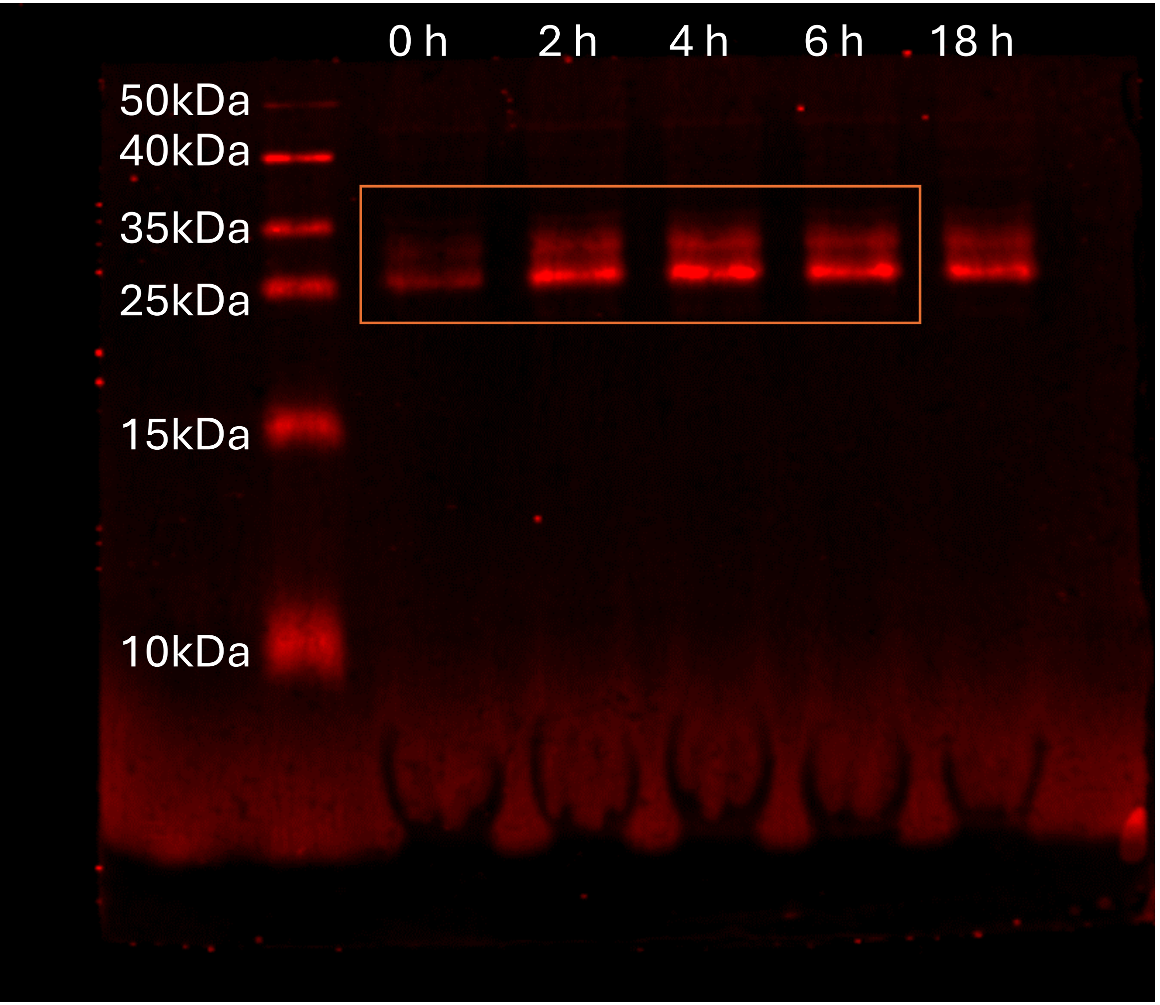
0 h
2 h
4 h
6 h
18 h
50kDa
40kDa
35kDa
25kDa
15kDa
10kDa

Actin

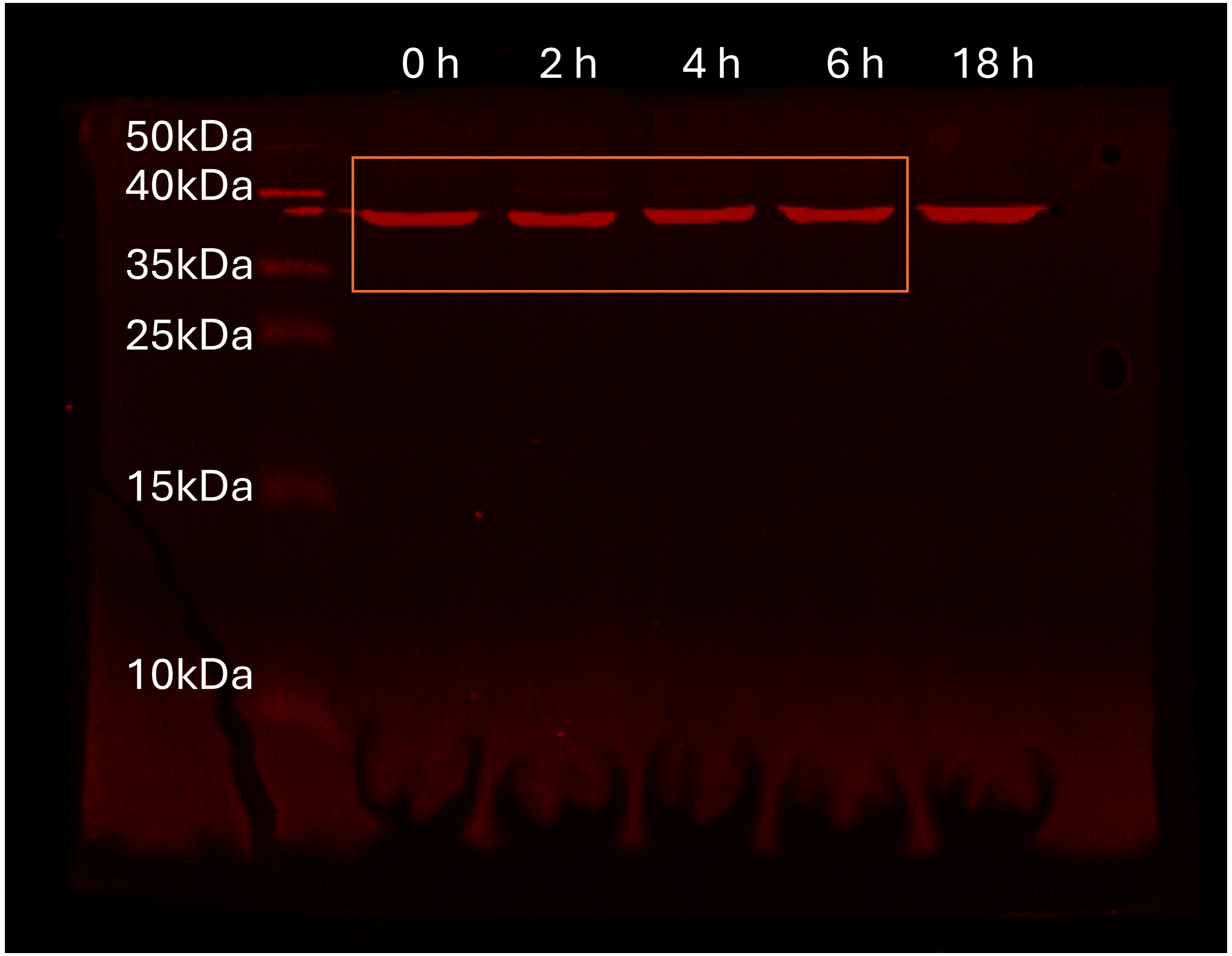
0 h
2 h
4 h
6 h
18 h
50kDa
40kDa
35kDa
25kDa
15kDa
10kDa

SYT10

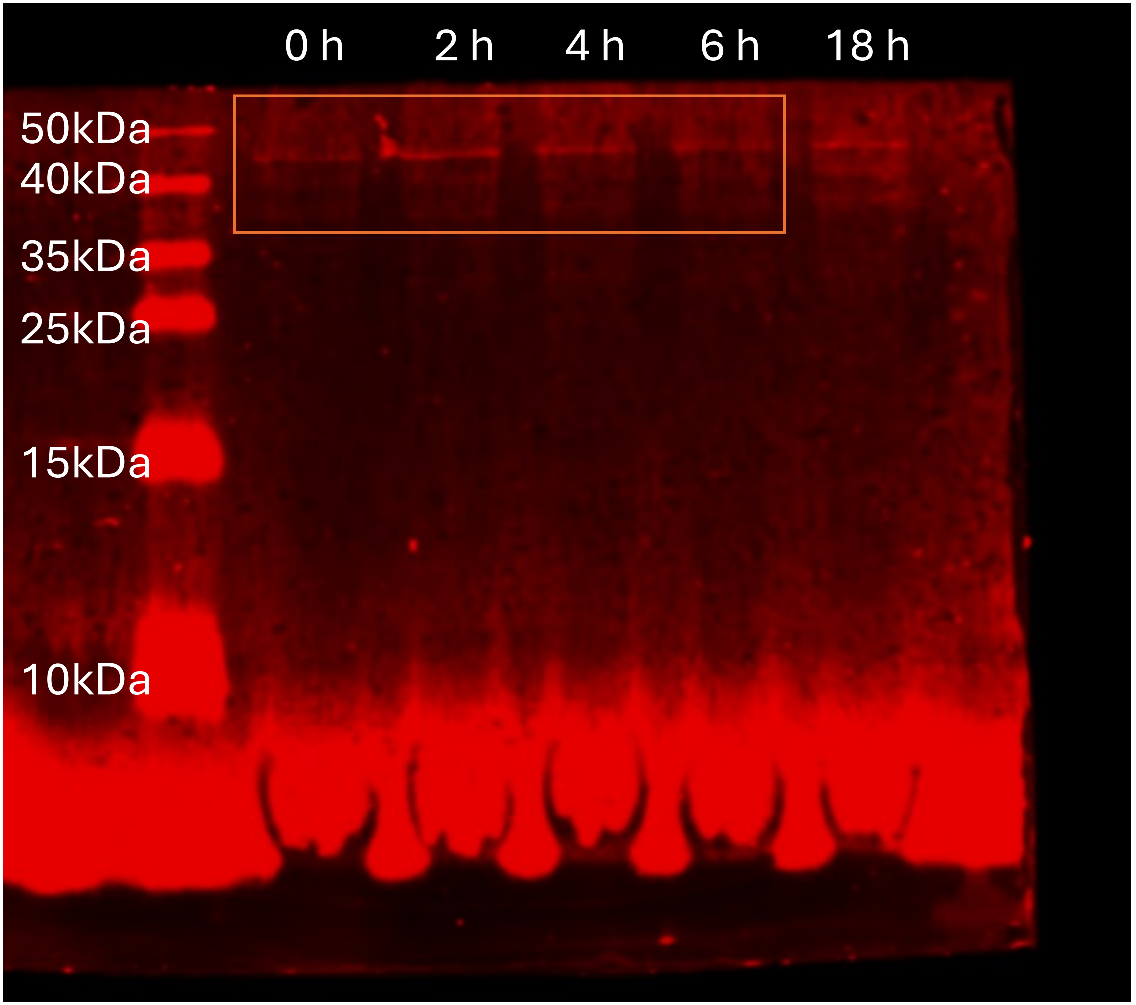
0 h
2 h
4 h
6 h
18 h
50kDa
40kDa
35kDa
25kDa
15kDa
10kDa

PIN1

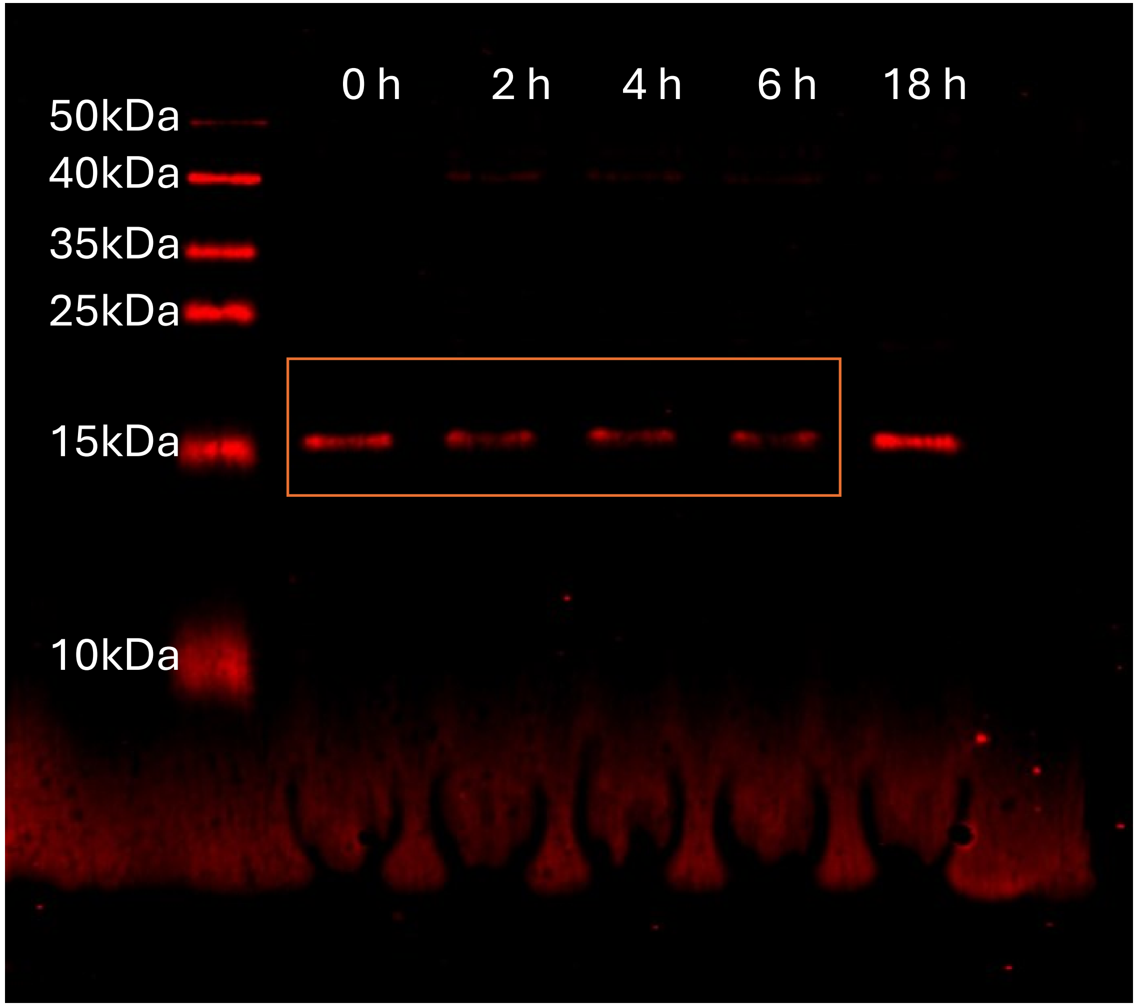
0 h
2 h
4 h
6 h
18 h
50kDa
40kDa
35kDa
25kDa
15kDa
10kDa
